# Comparisons Between Serum Levels of Hepcidin and Leptin in Male College-Level Endurance Runners and Sprinters

**DOI:** 10.3389/fnut.2021.657789

**Published:** 2021-05-31

**Authors:** Shinsuke Nirengi, Hirokazu Taniguchi, Aya Ishibashi, Mami Fujibayashi, Nao Akiyama, Kazuhiko Kotani, Kengo Ishihara, Naoki Sakane

**Affiliations:** ^1^Division of Preventive Medicine, Clinical Research Institute, National Hospital Organization Kyoto Medical Center, Kyoto, Japan; ^2^Department of Physiology and Cell Biology, Dorothy M. Davis Heart and Lung Research Institute, The Ohio State University Wexner Medical Center, Columbus, OH, United States; ^3^Division of Applied Life Sciences, Graduate School of Life and Environmental Sciences, Kyoto Prefectural University, Kyoto, Japan; ^4^Department of Life Sciences, The University of Tokyo, Tokyo, Japan; ^5^Faculty of Agriculture, Department of Food Science and Human Nutrition, Setsunan University, Osaka, Japan; ^6^Faculty of Agriculture, Ryukoku University, Shiga, Japan; ^7^Division of Community and Family Medicine, Center for Community Medicine, Jichi Medical University, Tochigi, Japan

**Keywords:** track and field, iron metabolism, adipokine, college athlete, body fat, diet

## Abstract

**Background:** Hepcidin-25 is a 25 amino acid hepatokine and a key regulator of iron metabolism related to iron deficiency anemia. Recent studies have suggested that an elevated hepcidin level is correlated with low energy availability. Leptin is an appetite-suppressing adipokine and has been reported to stimulate hepcidin production in animals and cultured cells. While leptin is modulated by exercise, it is known that endurance runners and sprinters practice different types of exercise. This study investigated and compared the relationships between hepcidin and leptin levels, iron status, and body fat to understand better the risk of iron deficiency anemia in endurance runners and sprinters.

**Methods:** Thirty-six male college track and field athletes (15 endurance runners and 21 sprinters) were recruited for this study. Dietary intake, body composition, and blood levels of ferritin, hepcidin-25, leptin, and adiponectin were measured. Correlations between hepcidin levels and ferritin, body fat, leptin, and adiponectin were evaluated using Pearson's correlation coefficient for each group.

**Results:** The endurance runners had lower hepcidin levels and higher leptin and adiponectin levels compared with sprinters. Ferritin was positively correlated with hepcidin-25 levels in both the endurance and sprinter groups. A positive correlation was observed between hepcidin-25 and body fat or leptin levels only in sprinters.

**Conclusion:** This is the first study investigating the relationship between blood levels of hepcidin and leptin in athletes. The positive correlation between hepcidin-25 and leptin was observed in sprinters but not endurance runners.

## Introduction

It is well-known that iron deficiency anemia is a problem for exercise performance and health in athletes (1–3). Therefore, it is highly desirable to monitor iron deficiency in athletes in the field. Hepcidin is secreted from the liver and adipose tissue and is a primary regulator of iron metabolism (4, 5). Excess hepcidin degrades the ferroportin export channels on the surface of macrophages and the intestinal duodenum, resulting in reduced iron recycling and absorption from the intestine (4, 5). The relationships between increased hepcidin levels and risk of iron deficiency have been investigated in various health settings, and hepcidin has been suggested as a surrogate marker for iron metabolism in athletes (4–9).

Excessive exercise increases the risk of iron deficiency (3), and multiple studies have attempted to clarify the relationship between blood levels of hepcidin and energy availability (7–10). The appetite-related hormone leptin is a well-known adipokine (11, 12) reported to stimulate hepcidin production in mice and cultured cells (13, 14). Exercise training modulates leptin levels and sensitivity, as well as appetite (15–17), and leptin-induced modulation of hepcidin may affect energy availability. Leptin is lower in body fat during fasting and acute exercise, which generally depicts the state of endurance runners (18). On the other hand, eating disorders were observed more in endurance runners than other types of athletes (19), which potentially disrupt leptin dynamics (20). However, no studies have investigated the relationship between hepcidin and leptin levels in athletes.

The type of exercise performed by athletes differentially exercise time, body fat, and hormone secretion, including leptin (15–17). In track and field clubs, endurance runners and sprinters typically engage in different types of exercise. Therefore, this study investigated the relationships between hepcidin and leptin, iron status, and body fat to understand better the risk of iron deficiency anemia in endurance runners and sprinters. We hypothesize that leptin is correlated with hepcidin level in sprinters but not in endurance runners.

## Materials and Methods

### Participants

Thirty-six male University track and field athletes (15 endurance runners and 21 sprinters) were enrolled in this cross-sectional study. The inclusion criterion was endurance runner or sprinter, and the exclusion criteria were the athletes who have iron deficiency (ferritin <20.0 ng/mL, one sprinter) (1). All athletes belonged to the same University club, and the team was in the top tier of University teams in Japan, running 5,000 m in <16 min and 30 s, and 100 m in <11.5 s. The athletes practiced 5–6 days per week, 3 h per day. This study was carried out in accordance with the recommendations of the Declaration of Helsinki (Fortaleza 2013). The study protocol was approved by the Ethics Committee of the Institutional Review Board of Kyoto Medical Center (approval number: 2013-005), and all participants provided written informed consent. The subjects were instructed to fast for 10 h and avoid exercise for 24 h before taking the following measurements.

### Body Composition

Body weight, body fat content, and skeletal muscle mass were measured using an Inbody 720 analyzer (Biospace, Seoul, Korea). The body fat and skeletal muscle were estimated using the impedance method by measuring the voltage drop initiated from a current as it passes between electrodes (21, 22).

### Questionnaire of Track and Field History and Dietary Record

Track and field history was assessed via a questionnaire. Daily intake of energy and nutrients was determined using the brief-type self-administered diet history questionnaire (BDHQ) (23, 24). The BDHQ is a four-page, fixed-portion questionnaire that estimates the dietary intake of 46 food and beverage items during the past month. Details of the BDHQ, including the food and beverage items queried and the methods used, are provided elsewhere (23, 24).

### Blood Analysis

Blood samples were collected from the antecubital vein between 9:30 and 10:30 am. Peripheral blood tests were performed to determine red blood cell (RBC) counts, hemoglobin (Hb) and hematocrit (Hct) levels, mean corpuscular volume, mean corpuscular hemoglobin, and mean corpuscular hemoglobin concentration. To quantify the active form of hepcidin, hepcidin-25, serum samples were mixed with synthetic human hepcidin (Peptide Institute, Osaka, Japan) as an internal standard and applied to a reverse-phase PLRP-S column (5 mm, 300 Å, 150 × 3 × 2.1 mm; Varian, Inc, Palo Alto, CA, USA). Levels of hepcidin-25 in the eluate were measured using a 4000 QTRAP liquid chromatography tandem mass spectrometry system (Applied Biosystems, Foster City, CA) (25). A LABOSPECT 008α automatic colorimetric analyzer (Hitachi High-Technologies Corporation, Schaumburg, IL, USA) was used to measure serum levels of iron. To test levels of iron stored in the body, we measured the concentrations of ferritin in serum using an iatro ferritin kit (LSI Medience Corporation, Tokyo, Japan). The leptin levels were quantified using a human leptin radioimmunoassay kit (EMD Millipore Corporation, Billerica, MA) (26). The adipokine adiponectin was measured using a human adiponectin latex kit (LSI Medience Corporation) (27).

### Statistical Analysis

Data were expressed as means ± SD. The normal distribution was tested using the Shapiro-Wilk test. The hepcidin-25 level was log-transformed since it is not normal distribution in the endurance runner group. Differences between groups were analyzed using the independent Student's or Mann- Whitney U test. The effect size was calculated by cohen's *d* value. The correlation analysis was used in a Pearson's correlation coefficient, respectively. A multivariate regression analysis was performed to evaluate the relationship between the log hepcidin-25 and leptin or body fat, independent of ferritin levels. A *P*-value < 0.05 was considered statistically significant. All statistical analyses were performed using SPSS Statistics software, version 22.0 (IBM Corp., Armonk, NY, USA).

## Results

The anthropometric characteristics of the study subjects are shown in Table 1. The participants had an average age of 20.1 ± 0.2 years and had engaged in track and field events for an average of 7.8 ± 0.3 (range, 4–11) years. As expected from previous studies (28, 29), the sprinters had a significantly higher body weight, body mass index, and lean mass and body fat compared with endurance runners (*P* < 0.05). There were no significant differences between endurance runners vs. sprinters for absolute intake of energy (2,387 ± 85.5 vs. 2,209 ± 136 kcal/day; *P* = 0.34), protein (81.3 ± 3.7 vs. 81.9 ± 5.7 g/day; *P* = 0.94), fat (68.7 ± 4.3 vs. 64.3 ± 4.2 g/day; *P* = 0.48), carbohydrates (336.6 ± 11.5 vs. 311.6 ± 25.0 g/day, *P* = 0.45), and iron (8.1 ± 2.0 vs. 8.6 ± 3.0 mg/day; *P* = 0.64). Anemia-related blood test parameters are shown in Table 2. As previously reported (1, 30, 31), endurance runners had lower concentrations of ferritin and Hb, numbers of RBCs, and % Hct compared with sprinters (*P* < 0.05).

**Table 1:** Comparison anthropometric characteristics between endurance runners and sprinters.

**Parameters**	**Endurance runners (*n* = 15)**	**Sprinters (*n* = 21)**	***P*-value**
Age, years	20.6 ± 0.4	20.6 ± 0.2	0.95
No. years played	7.8 ± 2.1	7.9 ± 1.5	0.93
Height, cm	169.2 ± 3.5	170.9 ± 7.0	0.39
Body weight, kg	55.1 ± 3.2	63.1 ± 5.7	<0.01
BMI, kg/m^2^	19.3 ± 1.2	21.6 ± 1.2	<0.01
Body fat, kg	5.2 ± 1.7	6.7 ± 1.8	0.02
Body fat, %	9.4 ± 2.9	10.6 ± 2.8	0.23
Skeletal muscle, kg	28.0 ± 1.5	32.2 ± 3.2	<0.01

*BMI, body mass index*.

**Table 2:** Comparison anemia related parameters between endurance runners and sprinters.

**Parameters**	**Endurance runners**	**Sprinters**	***P*-value**
	**(*n* = 15)**	**(*n* = 21)**	
Fe, mg/dL	112.2 ± 13.5	128.9 ± 8.4	0.28
Ferritin, ng/mL	55.7 ± 7.2	97.3 ± 6.7	<0.01
RBCs × 10^4^/mL	492.0 ± 8.4	533.5 ± 6.2	<0.01
Hb, g/dl	15.0 ± 0.3	16.2 ± 0.2	<0.01
Hct, %	45.5 ± 0.7	48.9 ± 0.6	<0.01
MCV, fL	92.9 ± 1.0	91.8 ± 0.7	0.27
MCH, pg	30.5 ± 0.2	30.3 ± 0.2	0.60
MCHC, %	32.9 ± 0.3	33.1 ± 0.2	0.60

*RBC, red blood cell; Hb, hemoglobin; Hct, hematocrit; MCV, mean corpuscular volume; MCH, mean corpuscular hemoglobin, MCHC, mean corpuscular hemoglobin concentration*.

**Figure 1:**
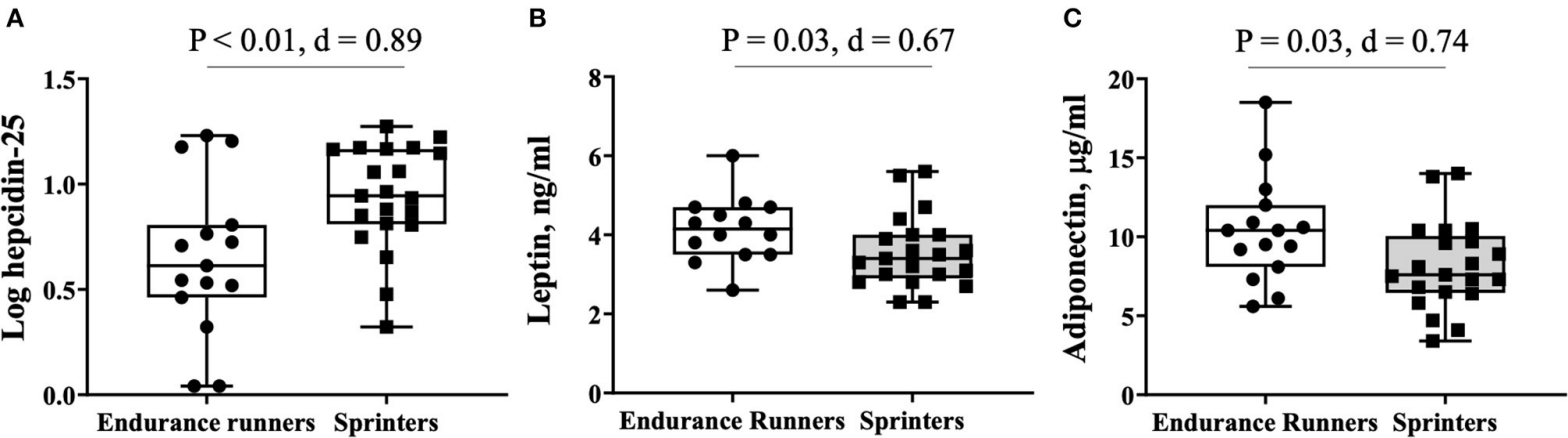
Comparison of hepatokine and adipokine levels between endurance runners and sprinters. **(A)** log hepcidin-25, **(B)** leptin, **(C)** adiponectin.

Figure 1 shows the serum levels of the hepcidin-25 and adipokines leptin and adiponectin in the endurance runner and sprinter groups. The serum levels of hepcidin-25 varied (range, 1.1–18.8 ng/ml) even though the participants belonged to the same club. The hepcidin-25 level (6.1 ± 5.3 vs. 9.9 ± 4.7 ng/ml; *d* = 0.76, *P* = 0.03), and log-hepcidin-25 levels (0.65 ± 0.37 vs. 0.93 ± 0.25 ng/ml; *d* = 0.89, *P* < 0.01) were lower in the endurance runners compared with sprinters. Leptin levels were higher in endurance runners compared with sprinters (4.2 ± 0.9 vs. 3.6 ± 0.9 ng/ml; *d* = 0.67, *P* = 0.03), and adiponectin levels (10.4 ± 3.4 vs. 8.1 ± 2.8 ng/ml; *d* = 0.74, *P* = 0.03) between these groups.

**Figure 2:**
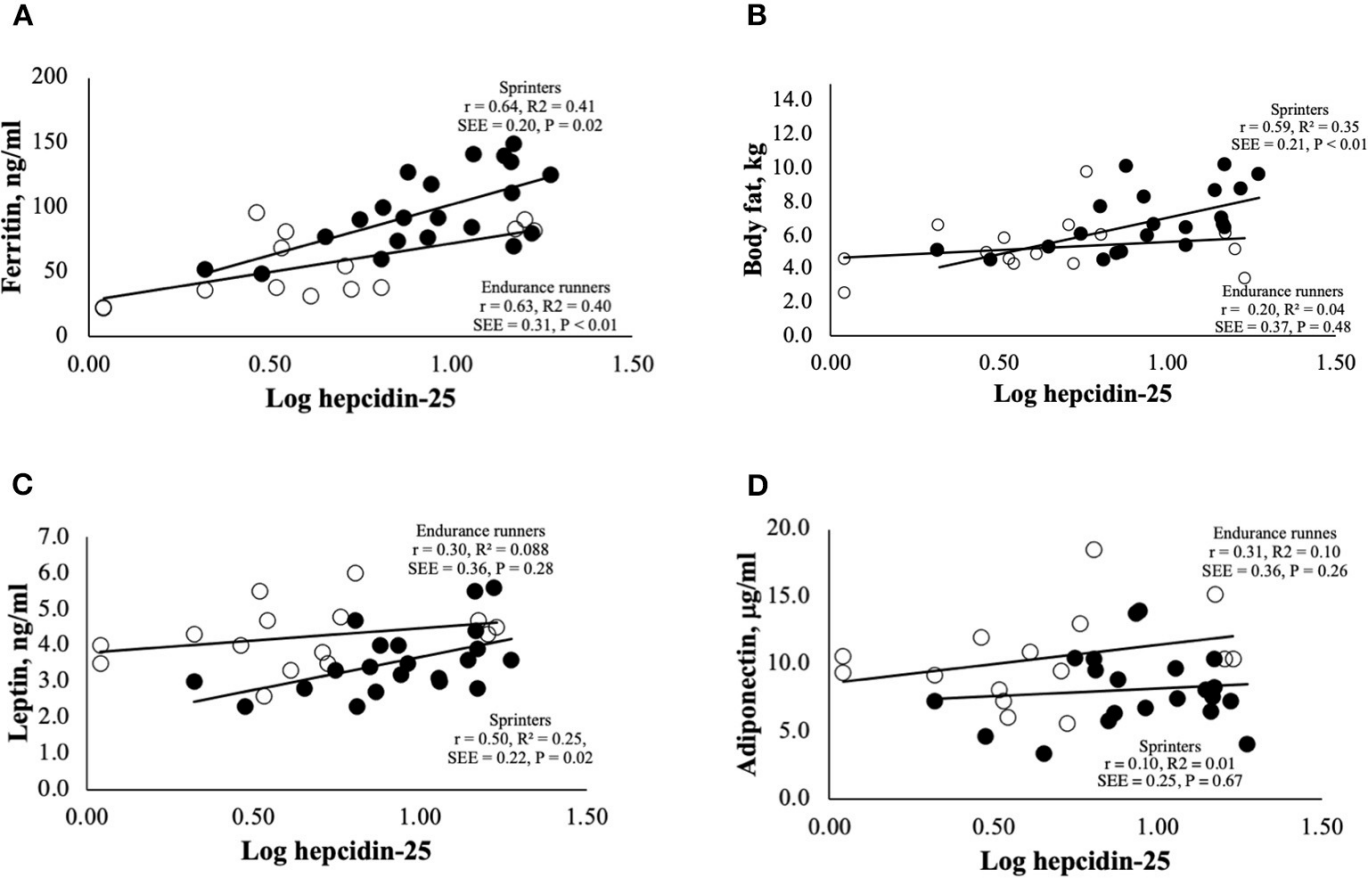
Correlations between serum levels of hepcidin-25 and **(A)** ferritin, **(B)** body fat, **(C)** leptin, and **(D)** adiponectin for endurance runners and sprinters. The hepcidin-25 value was log transformed, to keep normal distribution. Correlations were determined using Pearson's correlation coefficient. The open and closed circles represent the endurance runners and sprinters, respectively. SSE, Standard error of estimate.

Correlation analyses were performed to examine the relationship between hepcidin levels and adipokine levels (Figure 2). It was reported that hepcidin-25 levels were significantly and positively correlated with the amount of body fat and serum ferritin levels (5, 32, 33). Therefore, we investigated the relationship between hepcidin-25 and these parameters in endurance runners and sprinters. Levels of serum ferritin and hepcidin-25 were positively correlated in both the endurance runner and sprinter groups (Figure 2A). A positive correlation between hepcidin-25 and body fat was observed in sprinters but not in the endurance runners (Figure 2B). We investigated the relationship between serum levels of leptin and hepcidin-25 because leptin has been shown to correlate with body fat and appetite and stimulate hepcidin production (11–14). A significant correlation between leptin and hepcidin concentrations was observed in sprinters but not in endurance runners (Figure 2C), while there was no correlation observed between adiponectin and hepcidin in either group (Figure 2D). Furthermore, multivariate regression analysis showed that the leptin levels were correlated with hepcidin-25 levels independent of ferritin in sprinters but not observed in endurance runners (Table 3).

**Table 3:** Hepcidin-25 level is correlated with leptin and body fat in sprinters but not in endurance runners.

	**β**	**Standardized β**	***P***
**Log hepcidin-25 (Endurance runners)** ***r*** **=** **0.68**, ***R***^**2**^ **=** **0.46**, ***P*** **=** **0.03**			
Constant	−0.30	–	0.51
Ferritin	0.009	0.62	0.018
Leptin	0.11	0.25	0.28
**Log hepcidin-25 (Endurance runners)** ***r*** **=** **0.65**, ***R***^**2**^ **=** **0.32**, ***P*** **=** **0.046**			
Constant	−0.14	–	0.74
Ferritin	0.009	0.62	0.019
Body fat	0.058	0.18	0.45
**Log hepcidin-25 (Sprinters)** ***r*** **=** **0.78**, ***R***^**2**^ **=** **0.61**, ***P*** **<** **0.01**			
Constant	0.028	–	0.88
Ferritin	0.005	0.60	<0.01
Leptin	0.12	0.45	<0.01
**Log hepcidin-25 (Sprinters)** ***r*** **=** **0.77**, ***R***^**2**^ **=** **0.59**, ***P*** **<** **0.01**			
Constant	0.12	–	0.47
Ferritin	0.004	0.52	<0.01
Body fat	0.060	0.44	0.01

## Discussion

The main finding of the present study was that we discovered a correlation between the serum levels of hepcidin and leptin in male college-level sprinters but not endurance runners. This is the first study to show a relationship between hepcidin and leptin concentrations in athletes.

Although the mechanism underlying the association between hepcidin and leptin levels in the blood is unknown, there have been mouse and cell culture studies that indicated a relationship between these two proteins (13, 14). Serum hepcidin levels are low in leptin-deficient (ob/ob) mice. Interestingly, leptin receptor-deficient (db/db) mice have higher leptin levels than ob/ob mice, but low hepcidin levels (13), which suggests that leptin receptors may stimulate hepcidin production or secretion. Indeed, the administration of recombinant leptin to ob/ob mice for 2 weeks showed a significant increase in the levels of serum hepcidin and hepatic hepcidin mRNA (*Hamp*) in the liver (13). Furthermore, leptin stimulation increased hepcidin mRNA in cultured human HuH7 hepatoma cells (14).

The relationship between hepcidin and leptin levels observed in the sprinter group may be due to the stimulation of the leptin receptor. However, in the present study, there was no correlation between serum levels of hepcidin and leptin in endurance runners. Basically, endurance runners have a higher energy expenditure during daily training (e.g., 10–20 km of running/day, ~500–1,000 kcal/day) than sprinters. Besides, the practice without diet tends to be higher observed in long-distance runners (34). Since we did not estimate accurate dietary intake (e.g., 3-day weighted-food records) and energy expenditure, we did not estimate the energy valance or availability. However, compared to sprinters, a lower body fat amount, BMI, and anemia-related parameters (35, 36) in endurance runners may indicate the lower energy availability. In athletes, low energy availability may cause iron deficiency anemia (1–3, 8). The chronic stress from excessive exercise may disrupt leptin metabolism (15, 37–40). Leptin levels are not only decreased during chronic changes in body fat (37) but also during short-term changes corresponding to energy states (38–40), such as fasting and dietary restriction. In terms of iron metabolism, it was reported that the treatment of 3T3L-1 adipocytes with iron decreased the expression of leptin mRNA (37). Our data showed that endurance runners had lower iron status and higher leptin levels compared with sprinters regardless of the amount of body fat. Therefore, it is speculated that there was no relationship between the hepcidin and leptin levels in endurance runners because of disruption of energy and iron status induced by vigorous endurance-related exercise.

There were some limitations to this study. First, we did not evaluate leptin receptor sensitivity in our subjects. However, there is no way to evaluate the tissue leptin receptor in humans. Second, we did not evaluate exercise activity and did not estimate total energy intake using BDHQ; therefore we could not estimate energy availability. The doubly labeled water method would be needed to investigate detailed energy expenditure in a day. We evaluated dietary intake using a questionnaire. However, this method may not be appropriate for individual athletes. Indeed, according to our BDHQ data, our subjects consumed ~2,000 kcal/day of energy, which is the average recommended intake for a sedentary individual. To investigate the relationship between hepcidin levels and energy state, 3-day food intake records will be needed in future studies.

In conclusion, the positive correlation between serum levels of hepcidin-25 and leptin in sprinters and lack of correlation in endurance runners. Further studies are warranted to investigate the relationships between blood leptin and hepcidin levels and energy availability in athletes. This will provide useful insight on how to prevent sport-related anemia through hepcidin level monitoring.

## Data Availability Statement

The raw data supporting the conclusions of this article will be made available by the authors, without undue reservation.

## Ethics Statement

The studies involving human participants were reviewed and approved by Ethics Committee of the Institutional Review Board of Kyoto Medical Center. The patients/participants provided their written informed consent to participate in this study.

## Author Contributions

SN and NS designed the study. SN, HT, NA, KI, and NS collected and assembled of data. SN performed the statistical analysis and prepared the manuscript. HT, AI, MF, KK, KI, and NS did the trial management and helped to draft the manuscript with its critical review. All authors are in agreement with the manuscript and declare that the content has not been published elsewhere.

## Conflict of Interest

The authors declare that the research was conducted in the absence of any commercial or financial relationships that could be construed as a potential conflict of interest.
